# Golgi anti-apoptotic protein: a tale of camels, calcium, channels and cancer

**DOI:** 10.1098/rsob.170045

**Published:** 2017-05-03

**Authors:** Guia Carrara, Maddy Parsons, Nuno Saraiva, Geoffrey L. Smith

**Affiliations:** 1 Department of Pathology, University of Cambridge, Cambridge, CB2 1QP, UK; 2 Randall Division of Cell and Molecular Biophysics, King's College London, London SE1 1UL, UK; 3 CBIOS, Universidade Lusófona Research Centre for Biosciences and Health Technologies, Campo Grande 376, Lisbon 1749-024, Portugal

**Keywords:** Golgi anti-apoptotic protein, ion channel, calcium flux, cell adhesion and migration, Bax inhibitor-1, TMBIM

## Abstract

Golgi anti-apoptotic protein (GAAP), also known as transmembrane Bax inhibitor-1 motif-containing 4 (TMBIM4) or Lifeguard 4 (Lfg4), shares remarkable amino acid conservation with orthologues throughout eukaryotes, prokaryotes and some orthopoxviruses, suggesting a highly conserved function. GAAPs regulate Ca^2+^ levels and fluxes from the Golgi and endoplasmic reticulum, confer resistance to a broad range of apoptotic stimuli, promote cell adhesion and migration via the activation of store-operated Ca^2+^ entry, are essential for the viability of human cells, and affect orthopoxvirus virulence. GAAPs are oligomeric, multi-transmembrane proteins that are resident in Golgi membranes and form cation-selective ion channels that may explain the multiple functions of these proteins. Residues contributing to the ion-conducting pore have been defined and provide the first clues about the mechanistic link between these very different functions of GAAP. Although GAAPs are naturally oligomeric, they can also function as monomers, a feature that distinguishes them from other virus-encoded ion channels that must oligomerize for function. This review summarizes the known functions of GAAPs and discusses their potential importance in disease.

## 1. Introduction: the transmembrane BI-1-containing motif/Lifeguard family and the ancestral GAAP

### 1.1. Discovery and origins of GAAPs

In 2002, sequencing of the camelpox virus (CMLV) genome identified a novel gene, *6L*, located in the left terminal region of the genome [1]. This gene encodes a highly hydrophobic, 237 amino acid (aa), membrane protein of approximately 23 kDa that was characterized and named Golgi anti-apoptotic protein (GAAP) based on its intracellular localization and its first described function [2]. Bioinformatic analysis identified GAAP relatives in some other orthopoxviruses, including some strains of vaccinia virus (VACV) [1], the vaccine used to eradicate smallpox, but also throughout higher eukaryotes, as well as some sponges, fungi, yeast and an increasing number of prokaryotes [2–5]. Similarly, a human orthologue of unknown function was identified from the human genome sequencing project, and was expressed and characterized [2].

Since the discovery of GAAPs, the viral and human versions (vGAAP and hGAAP, respectively) have been the most studied, leading to the identification of several cellular functions and structural properties of GAAPs. Both vGAAP and hGAAP localize to the Golgi apparatus [2] and confer resistance to a broad range of pro-apoptotic stimuli of both intrinsic and extrinsic origins [2,4]. Human GAAP regulates the Ca^2+^ content and fluxes from the principal intracellular Ca^2+^ stores (Golgi and endoplasmic reticulum, ER) [6] and promotes cell adhesion and migration via the activation of store-operated Ca^2+^ entry (SOCE) from the extracellular space [7]. GAAPs are multi-transmembrane proteins that are also known to homo-oligomerize, and vGAAPs from VACV and CMLV were shown to form cation-selective ion channels, potentially forming the basis for the modulation of its diverse functions [4,8].

### 1.2. Human GAAP: a housekeeping gene essential for cell survival

hGAAP mRNA is expressed ubiquitously across all human tissues tested, with lower expression detected in the brain compared to other tissues [2,9,10]. The conservation of GAAP expression suggested that its function may be fundamental to the function of a wide variety of cell types. Indeed, hGAAP was proposed to be an essential, universal, housekeeping protein based on microarray analyses [11], and knockdown of endogenous hGAAP by small interfering RNA (siRNA) demonstrated that it is essential for cell viability because cells die by apoptosis in its absence [2]. Interestingly, the viral protein is sufficiently similar (73% aa identity) to complement for loss of hGAAP in human cells. The requirement of other GAAP relatives in simpler systems such as yeast or bacteria remains to be assessed but may provide interesting information on the importance and conservation of its essential housekeeping functions.

### 1.3. Why do some orthopoxviruses express a viral GAAP?

Viral GAAPs are expressed by CMLV, a few strains of VACV and cowpox virus, and are non-essential for viral replication [2]. However, it affects virus infection *in vivo* and deletion of vGAAP from VACV strain Evans caused an increase in virus virulence *in vivo* accompanied by an increased infiltration of leucocytes into infected tissue [2]. Given that mammalian cells express a GAAP, why have some orthopoxviruses evolved to express a vGAAP? Possible explanations are (i) that the viral protein has subtly different properties to the cellular protein and these are advantageous to the virus, (ii) that the induction of cell motility by vGAAP is beneficial to virus spread, (iii) that vGAAP regulates the host response to infection, and (iv) that the level of expression of cellular GAAP in mammalian cells is low, and so expression at higher levels, as observed during virus infection [2], is beneficial. The low level of cellular GAAP expression will be reduced further during infection because orthopoxviruses like VACV induce a rapid shut off of cellular protein synthesis [12] mediated by the de-capping enzymes D9 and D10 [13–15] and protein 169 [16]. Therefore, the expression of vGAAP may help keep the infected cells more suitable hosts to support virus replication. However, under the cell culture conditions tested, a VACV engineered to not express vGAAP replicated as well as control viruses expressing vGAAP [2]. Given that loss of vGAAP from VACV strain Evans affects virus virulence and the influx of inflammatory cells into infected tissue [2], vGAAP can be added to the long list of immuno-regulators expressed by VACV that affect the host response to infection [17].

### 1.4. GAAPs within the TMBIM and Lifeguard family: an evolutionary perspective

Initially, GAAP was classified as the fourth member of the transmembrane Bax (Bcl-2-associated X protein) inhibitor-1 motif-containing (TMBIM) family, based on similarities in the number of predicted transmembrane domains (6–7 TMDs), known as the UPF0005 motif, and a shared anti-apoptotic function with the most studied member of the family, Bax inhibitor-1 (BI-1), from which the family name was derived [18–20]. Currently, this family includes seven well-conserved members: responsive to centrifugal force and shear stress gene 1 protein (RECS1) (TMBIM1), TMBIM1b, FAS inhibitory molecule 2 (FAIM2)/LFG (TMBIM2), glutamate receptor ionotropic NMDA-associated protein (GRINA) (TMBIM3), GAAP (TMBIM4), growth hormone-inducible transmembrane protein (Ghitm) (TMBIM5) and BI-1 (TMBIM6) [2,20–22]. Although the TMBIM family has been the most commonly used classification, a subsequent phylogenetic analysis showed that five of its members, including GAAP, cluster independently of the Bax-motif-containing proteins, Ghitm and BI-1, as far back as the root of all animals and possibly extant eukaryotes, thus creating a diverging family nomenclature known as the Lifeguard (LFG) family [5,21]. Current LFG family members are therefore GRINA (Lfg1), FAIM2/LFG (Lfg2), RECS1 (Lfg3), GAAP (Lfg4) and TMBIM1b (Lfg5). GAAP contains key sequence similarities and differences of functional consequence with both LFG and BI-1 families. For instance, the SPE[ED]Y motif between TMD6 and hydrophobic loop 7 of GAAP, which is central within the channel pore and important for cell adhesion and migratory functions, is present throughout the LFG family members but absent from Ghitm and BI-1 [4,5,21]. Conversely, a series of charged residues at the C terminus of GAAP (LEAVNKK) is conserved only in BI-1 (EKDKKKEKK), Ghitm (RKK) and to a minimal extent GRINA (KE) [4], with similar critical requirements for GAAP and BI-1 in regulating cell adhesion, apoptosis and Ca^2+^ homeostasis [3,7,23–25]. Furthermore, the most closely related members to GAAPs are LFG protein and BI-1 with 34 and 28% aa identity, respectively [2]. Despite the fact that the relationship between these proteins is unclear, it is evident that a divergence exists within the TMBIM family. If this separation is as ancient as proposed, the LFG family would constitute an independent family from Ghitm and BI-1, rather than a subclass of the TMBIM family. Nevertheless, phylogenetic analysis from both classification systems indicate the most probable family progenitor to have been a GAAP-like ancestor that expanded by a series of duplication and subsequent modification events to generate the current TMBIM and LFG family members [5,19,21]. This could explain why GAAP and BI-1 share some properties, such as the charged C terminus, that is absent in some other family members. More specifically, expansion of the five LFG family members from a GAAP-like progenitor was dated by phylogenetic analysis as prior to the divergence of plants and protozoa about 2000 million years ago [5,21].

TMBIM and LFG family members lack clear functional motifs indicative of function [4,21], and therefore family members have been studied independently, and findings from one member have often been tested and applied in the study of other members. Given the high degree of conservation between these proteins, this exercise has been productive, highlighting many similarities as well as functional variations (table 1). Subsequent studies were aimed at reassembling the known functions of TMBIM/LFG family members [18,19,21,67,68]. Indeed, a recent review proposed a global ‘stress integrator’ or ‘sentinel’ role for the family, thus regulating multiple essential adaptive responses to global stresses caused by environmental changes across different interconnected signalling pathways and tissues [18].

**Table 1.** Summary of functional, structural, tissue expression and evolutionary information currently available from the literature regarding members of the TMBIM and LFG family. All information relates to human unless otherwise mentioned. ATF4, activating transcription factor 4; BCL-2, B-cell lymphoma 2; BCL-X_L_, B-cell lymphoma—extra large; CMD, cystic medial degeneration; CNS, central nervous system; ER, endoplasmatic reticulum; FasL, Fas ligand; IP_3_R, inositol 1,4,5-trisphosphate receptor; KO, knockout; LFG, Lifeguard; MMP-9, metalloproteinase 9; NBL, neuroblastoma; NSCLC, non-small cell lung cancer; PERK, PRKR-like ER kinase; PM, plasma membrane; ROS, reactive oxygen species; SOCE, store-operated Ca^2+^ entry; TMD, transmembrane domain; TNFα, tumour necrosis factor alpha; TRIM21, tripartite motif-containing protein 21; UPR, unfolded protein response.

name	cellular localization	tissue expression	function/activity	topology structure	conservation*	human cancers
TMBIM1 (RECS1, Lfg3)	mostly Golgi [19,26] endosome and lysosome [21]	all but thymus, testis and spleen [21] muscle tissue in particular [19]	inhibits Fas ligand-induced apoptosis; reduces Fas trafficking to the PM without changing total Fas levels [26] downregulates aortic MMP-9 production and activity [27] aged TMBIM1-deficient mice are prone to CMD, a pathology frequently associated with aortic aneurysms [27,28] upregulated in response to mechanical stress [29]	six to seven predicted TMDs with uncertainty lying in the seventh hydrophobic region [3,26]	vertebrates, insects and nematodes	a new risk susceptibility SN associated with colorectal cancer was identified intronic to TMBIM1 [30]
TMBIM1b (Lfg5)	unknown [21]	unknown in human and testis in mice [5,21]	unknown	six predicted TMDs [20]	eutherian mammals [21]	
TMBIM2 (FAIM2, LFG, NMP35, Lfg2)	Golgi [19] plasma membrane lipid rafts [21,31]	predominantly central nervous system [9,19,21,31–33]	attenuates Fas ligand but not TNFα-induced apoptosis; binds to Fas receptor and interferes with caspase-8 activation [31,33,34] knockdown in the mouse CNS causes reduced cerebellar size, spontaneous activation of caspase-8 and caspase-3 in Purkinge cells, and cerebellum susceptibility to Fas-mediated cell death [35] reduced expression reduces cell adhesion, and enhances sphere growth, cell migration and metastasis of NBL cells [36] inhibits ER Ca^2+^ release in response to FasL stimulation [37]	seven predicted TMDs with uncertainty lying in the seventh hydrophobic region [3]	vertebrates, insects, nematodes and plants.	low *LFG* levels correlate with worse overall survival of NBL patients; in NBL cells, LFG expression is directly repressed by the MYCN oncogene at the transcriptional level [36] overexpression correlates with high primary breast tumours grades and reduced Fas sensitivity; over-expressed in several human breast cancer cell lines [38] knockdown renders MDA-MB-231 cells more susceptible to cisplatin treatment *in vitro*, possibly due to resulting Fas activation and increased caspase-8 activity [39] in MDA-MB-231 cells, TRIM21 interacts with LFG and has a repressive effect on LFG expression [40]
TMBIM3 (GRINA, NMDARP-71, OTMP, PM02, Lfg1)	Golgi [19,41] plasma membrane [21]	all, including central nervous system [9,19,21,42]	anti-apoptotic (not require for cell viability) under ER stress, UPR upregulates TMBIM3 levels in a PERK- (a UPR stress factor) and ATF4 (a transcription factor)-dependent manner [41] reduces IP_3_R-mediated ER Ca^2+^ release without altering ER Ca^2+^ content or passive leak by interacting with and downregulating IP_3_R activity [41] synergistic anti-apoptotic and ER Ca^2+^ homeostasis activity with BI-1 [41]	six to seven predicted TMDs with uncertain seventh hydrophobic region [3]	vertebrates, insects, nematodes, plants and yeast	
TMBIM4 (GAAP, Z-protein, Lfg4)	Golgi and some ER [2,3]	all, ubiquitous [2,9,19,21]	anti-apoptotic against intrinsic and extrinsic stimuli including TNF-α and FasL; required for cell viability [2,3,8] reduces basal Golgi and ER Ca^2+^ content and IP_3_R-mediated release in response to apoptotic stimuli [6] enhances cell adhesion and migration by stimulating Ca^2+^ influx across the plasma membrane via SOCE, thus modulating calpain 2 activation [7] ion channel (cation-selective) [4] oligomerizes in a pH-dependent manner monomer maintains its anti-apoptotic activity and its effect on Ca^2+^ homeostasis [8] housekeeping gene [11]	six TMDs with a C-terminal semi-hydrophobic loop [3,4]	vertebrates, insects, nematodes, fungi, plants, sponges, alveolates, yeast, bacteria and viruses.	upregulated in glioblastoma multiforme, which is associated with poor outcome [43] dysregulated in NSCLC, leading to hGAAP being proposed as a novel candidate prognostic marker for this disease in patients who have never smoked [44] enhances cell adhesion and migration in human osteosarcoma and cervical cancer cells [4,7]
TMBIM5 (Ghitm)	mitochondria inner membrane [45]	all [9,19]	inhibits mitochondrial fragmentation and apoptotic release of cyt c and Smac/Diablo [45]	six to seven predicted TMDs with uncertain seventh hydrophobic region [3]	vertebrates, insects and nematodes	
TMBIM6 (BI-1)	ER [3,19]	all, but predominantly in skeletal muscle, kidney, liver and spleen [9,19]	anti-apoptotic (intrinsic and ER stress, but not extrinsic FasL and TNFα) [22,24,46,47] interacts with Bcl-2 and Bcl-X_L_ [22] reduces basal ER Ca^2+^ content and release, by enhancing Ca^2+^ leakage from ER stores (pH-dependent) and interacting with and sensitizing IP_3_R [23,47,48] Ca^2^-permeable pH-sensitive ion channel, or a Ca^2+^/H^+^ antiporter-like activity [4,49–52] under low cytosolic pH, BI-1 Ca^2+^/H^+^ antiporter activity can become pro-apoptotic due to ER Ca^2+^ store depletion [23] participates in UPR by binding and inhibiting IRE1α [53] inhibits ROS production and promotes anti-oxidant HO-1 production [23,54] increases actin polymerization and SOCE and interacts with G-actin [25] BI-1 KO mouse indicates BI-1 plays a role in the adaptive immune system by regulating B- and T-cell function, and the intracellular Ca^2+^ homeostasis, SOCE and survival of immune cells [55]	six TMDs+a C-terminal semi-hydrophobic loop [4,49–52] seven complete TMDs (BsYetJ, BI-1 from bacteria) [52] crystal structure shows pore [52]	vertebrates, insects, fungi, plants, yeast and bacteria	upregulated in: glioma, lung adenocarcinoma, NSCLC, breast, prostate, uterine and ovarian cancers; down-regulated in some forms of stomach, colon, kidney, lung and rectal cancers [56–62] increases cancer progression and metastasis [59,63–65] overexpression of BI-1 enhances NIH3T3 cell transformation and tumourigenicity [65] overexpression increases glucose consumption, reduces O_2_ consumption and acidifies the extracellular space [63] BI-1 has been proposed as a target for anti-cancer therapeutics by anthracyclines [66]

*Conservation at the aa level.

The strong conservation of hydrophobicity profile within TMBIM and LFG members suggests that structure and/or hydrophobicity must be a critical aspect of their function [3,19]. This fits with the hypothesis that the ion channel activity of GAAP and BI-1 constitutes the core function from which their other functions are derived as downstream target effects.

### 1.5. Why is GAAP highly conserved?

**Figure 1.**
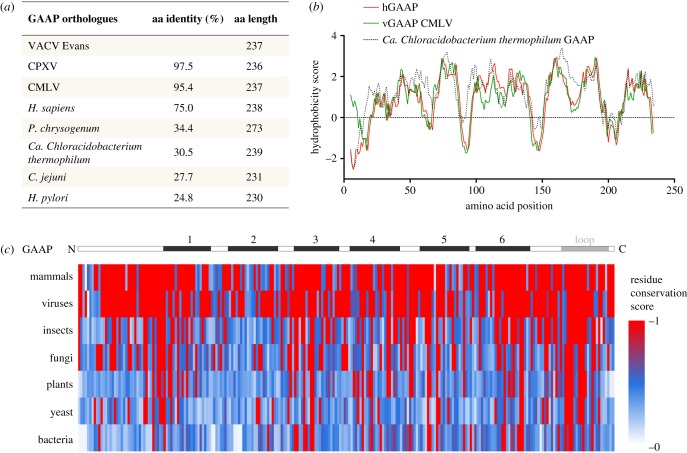
Conservation within the GAAPs. The extent of hydrophobicity and sequence conservation among GAAP orthologues. (*a*) The aa identities calculated by the BLASTP server and differences in aa length are indicated. (*b*) The hydrophobicity profile for hGAAP (eukaryote) was aligned with that of viral (vGAAP from CMLV) and prokaryotic (*Ca. Chloracidobacterium thermophilum*) GAAP representatives. Complete aa sequences were used for all. (*c*) aa sequence alignment of hGAAP against GAAP orthologues from two to three representative members from each taxon. The level of conservation for each residue was scored according to Scorecons and represented in a colour gradient, with red and white indicating identity and no similarity, respectively. Sequences analysed include *Homo sapiens*, *Bos taurus* and *Gallus gallus* (vertebrates); VACV Evans, CMLV and CPXV (viruses); *Cerapachys biroi* and *Tribolium castaneum* (insects); *Penicillium chrysogenum* and *Tuber melanosporum* (fungi); *Arabidopsis thaliana*, *Genlisea aurea* and *Zea mays* (plants); *Schizosaccharomyces pombe* and *Saccharomyces cerevisiae* (yeast); and *Campylobacter jejuni*, *Helicobacter pylori* and *Candidatus Chloracidobacterium thermophilum* (bacteria). Black and grey boxes indicate the location of TMDs 1–6 and the hydrophobic region/loop 7 of GAAPs, respectively. Adapted from Carrara *et al*. [4].

Orthologues of GAAP, identified throughout eukaryotes and recently in some prokaryotes, are characterized by a unique degree of protein sequence identity and length, with the majority of proteins differing by only 1–4 aa in length, and a strikingly conserved hydrophobicity profile maintained right down to bacteria (figure 1), suggesting an important fundamental cellular function, for which transmembrane structure is necessary [2,4]. Unlike in mammals, vGAAP is not ubiquitous among viruses and is found only in a subset of orthopoxviruses that include 3 of 16 strains of VACV examined, CMLV and cowpox virus [2,4]. In these viruses, the vGAAPs differ in only a few aa from one another and share more than 98% aa identity, and therefore also share the conserved membrane structure (figure 1) [4]. The level of conservation in aa length (1 aa difference), identity (73%) and hydrophobicity profile between vGAAPs and hGAAP is greater than many other VACV proteins that have a known mammalian orthologue [2]. For comparison, VACV strain Western Reserve proteins B15 [69] and B8 [70] share only 33% and 25% aa identity with the extracellular domain of the human interleukin-1β receptor and interferon-γ receptor, respectively. The much closer similarity of vGAAP and mammalian GAAPs might reflect a more recent acquisition of the host gene by an ancestral poxvirus, or conservation of GAAP sequence due to functional requirements. In this regard, the analysis of the nucleotide composition of the viral and mammalian GAAPs is informative. Orthopoxviruses such as VACV, cowpox virus and CMVL have genomes with high A+T content (67% for VACV) that differ considerably from that of humans and other mammalian genomes, and consequently the codon usage of these viruses also differs from their mammalian hosts [1,71]. But the A+T content of vGAAP from VACV (57.6%) or CMLV (59.0%) is more similar to hGAAP (57.5%) than it is to the majority of the virus genome (67%). So it has not yet adapted to the nucleotide composition of the virus genome as a whole, either due to relatively recent gene acquisition or due to functional constraints. The ability of VACV GAAP to restore cell viability following siRNA knock down of the essential endogenous hGAAP demonstrates that this conservation has retained protein function [2].

The high degree of conservation between GAAPs extends beyond other poxviruses and mammals to distant eukaryotes and even prokaryotes (figure 1). For instance, hGAAP shares 38% aa identity and 3 aa difference in length with GAAPs from *Arabidopsis thaliana* [2], and vGAAP is quite conserved with bacterial GAAPs including *Candidatus Chloracidobacterium thermophilum* and *Campylobacter jejuni* (30.5% or 27.7% aa identity, and only 2 or 6 aa difference in length, respectively), as well as a fungal GAAP in *Penicillium chrysogenum* (34.4% aa identity) [4]. This conservation is also strictly maintained in the hydrophobicity profiles of these GAAPs [4]. This remarkable conservation for such distantly related organisms supports a highly conserved ancestral structure and function [4]. Although GAAPs have not been reported so far in archaea, the extensive list of orthologues is expected to expand with the availability of newly sequenced genomes, particularly among prokaryotes. Considering that structure and activity of ion channels have evolved in prokaryotes long before the emergence of complex multicellular organisms [72], these novel and largely uncharacterized bacterial GAAPs merit study and may provide a simpler system, away from the complexities of inter-compartmentalized Ca^2+^ fluxes of organelles, from which to dissect GAAP core ion channel activity. Given the essential fundamental functions and ancestral origins of GAAP, testing the extent of conservation of its other functions down the evolutionary tree could provide answers to other fundamental and essential cellular pathways such as ion/Ca^2+^ flux and cell viability through apoptosis regulation.

## 2. GAAPs are Golgi ion channel proteins

### 2.1. GAAP ion channel activity, a core function

GAAP is predominantly a Golgi-resident protein [2]. This was demonstrated first with anti-GAAP antibody and immuno-electron microscopy [2] and then by expressing C-terminally tagged GAAPs [2–4,7,8]. However, as GAAP concentration increases during ectopic expression, hGAAP and vGAAPs also become detectable in the ER, presumably as a result of overexpression and Golgi saturation [2]. With time, this accumulation in the ER, a much larger organelle, is probably why GAAP localization has also been reported in the ER [18,67,73].

**Figure 2.**
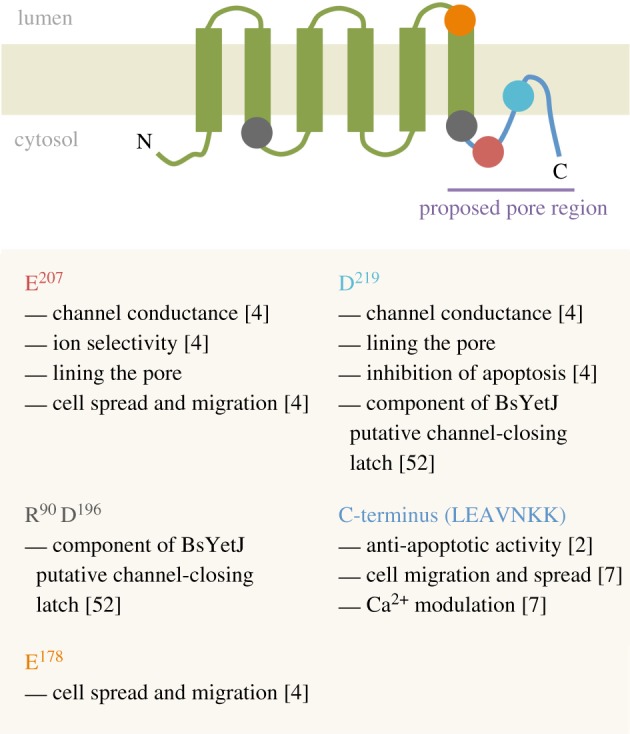
Diagram of GAAP highlighting regions/residues of importance for its different functions.

vGAAPs from VACV and CMLV form ion channels that are selective for cations [4]. This discovery remains the first report of an ion channel encoded by poxviruses. Electrophysiological characterization of single GAAP channels relied on spontaneous opening of these channels consistent with suggestions that the loss of Ca^2+^ from intracellular stores following hGAAP or hBI-1 expression is due to passive leakage [6,23]. GAAPs and hBI-1 inhibit apoptosis, increase cell spreading and migration speed and reduce the Ca^2+^ content of intracellular stores. Whether these effects are independent or the result of a common core function of these proteins is only now becoming clearer. Mutagenesis showed that two important biological effects of vGAAP, apoptosis and migration, are separable. Residues E^207^ or E^178^ are important for cell migration and adhesion but do not affect the ability of vGAAP to protect cells from apoptosis. In contrast, D^219^ is required for the anti-apoptotic activity of vGAAP but not cell migration and adhesion, but both functions are susceptible to mutation of the pore-associated residues E^207^ and D^219^ [4] (figure 2). This suggests that the ion channel activity of GAAPs may constitute the core function from which cell adhesion, migration and apoptotic protection are regulated. Consistent with the observations with GAAPs, mutation of the equivalent D^219^ residue in hBI-1 (D^213^ in hBI-1) attenuates the ability of BI-1 to reduce the Ca^2+^ content of the ER [51]. Furthermore, in the bacterial BI-1 orthologue, BsYetJ, this residue forms part of salt bridges that regulate pore opening upon protonation [52], suggesting that Ca^2+^ regulation and channel activity in BI-1 are also likely to be linked. However, the separation of major functions such as inhibition of apoptosis and cell migration has not been studied in BI-1. Despite having a topology that is broadly reminiscent of the α-subunits of eukaryotic voltage-gated channels [74], GAAPs lack obvious signature motifs related to selectivity or conductance [4]. This suggests that GAAPs may form channels with novel structures and may have a unique mechanism of action.

### 2.2. Consequences of GAAP oligomerization

Many ion channels are oligomeric proteins and oligomerization (homo or hetero) plays an important role in the regulation of ion flux or channel conductance. Most TMBIM proteins show several species with differing electrophoretic mobility during SDS-PAGE suggesting that homo-oligomerization is a conserved feature of this family [23,41,50]. Although a pH-dependent regulation mechanism has been demonstrated for multiple functions of BI-1, a pH-dependent mechanism for GAAP activity remains elusive. However, the oligomerization of vGAAP and hGAAP is dependent on pH, with a more alkaline pH favouring oligomerization. This is contrary to basal Golgi pH, which is more acidic than that of the ER. In the case of vGAAP, two cysteine residues responsible for direct protein oligomerization were identified [8]. Using Förster resonance energy transfer (FRET), it was shown that a vGAAP double cysteine mutant (^C9S/C60S^vGAAP) was unable to oligomerize in native Golgi membranes of live cells. Although no oligomerization was detectable by FRET, this mutant retained its anti-apoptotic activity and its effect on intracellular Ca^2+^ stores, proving that monomeric vGAAP is functional. Therefore, a model where the oligomeric state provides an on–off switch for GAAP activity is highly unlikely. However, an alteration of conductance or ion flux might be influenced by oligomerization and this remains to be tested electrophysiologically using the monomeric GAAP mutant. Surprisingly, the cysteine residues responsible for vGAAP oligomerization are absent from hGAAP, suggesting a different oligomerization mechanism for hGAAP that is independent of cysteines.

BI-1 and GAAP oligomerization are at least partially pH-dependent. An acidification of the cytosol results in increased oligomerization of BI-1, and BI-1-expressing cells show more Ca^2+^ release from stores under acidic conditions, but there is still no proof that the two are directly linked [23]. Amino acid residues required for BI-1 oligomerization have not been identified and so no monomeric BI-1 mutant is available to test if oligomerization is essential for its ion channel function. Although a BI-1 C-terminal peptide, which lacks all the regions identified in hGAAP that are required for oligomerization, is able to conduct ions across a membrane [75], its actual oligomeric state is unknown.

Interaction between different members of the TMBIM family has been reported, namely between BI-1 and GRINA, suggesting that a possible hetero-oligomerization of different TMBIM members could be relevant for TMBIM activity or regulation [41]. A limitation of this approach is the fact that most protein–protein interaction data were obtained from co-immunoprecipitation assays, and TMBIM proteins are highly susceptible to co-precipitate with any membrane protein or highly abundant proteins due to their high degree of hydrophobicity [4]. Even under very stringent conditions, it was possible to co-precipitate GAAP with several membrane and soluble proteins, some of which do not even localize at the Golgi. Therefore, no convincing protein interactions have so far been detected with any of the GAAPs. To address this issue, more robust techniques such as FRET [76], protein complementation assays [77] or two-hybrid assays [78] could be used to investigate TMBIM protein–protein interactions.

### 2.3. GAAP topology is unique among mammalian and viral ion channels

Owing to the highly hydrophobic nature of ion channels, which makes them experimentally difficult proteins to work with, the majority of channel structures have remained unsolved. The first ion channel structure solved was the prokaryotic KcsA K^+^ channel in 1998 [79], and thereafter ion channel structure has been a rapidly growing field.

**Figure 3.**
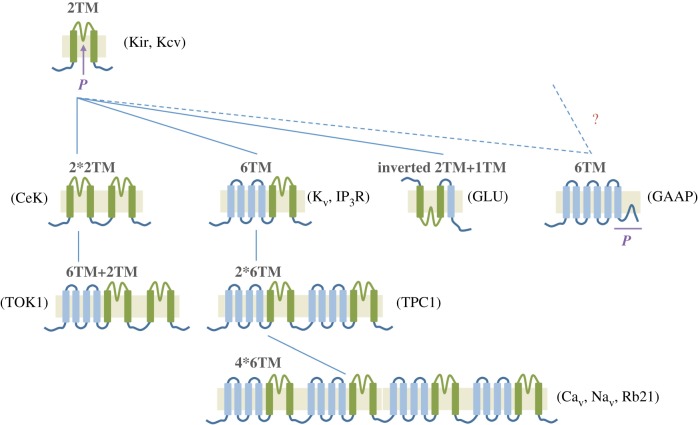
Current model of the structural phylogeny of voltage-gated K^+^ channels – where does GAAP fit in? The presumed precursor (green) consists of two transmembrane regions (*2TM*) separated by a pore-forming loop (*P*), which is common to all K^+^ channels. This *2TM* ancestor is expected to resemble the prokaryotic Kir and the viral Kcv. Other ion channels are thought to have arisen from the combination of duplication events such as 2*2TM, and the addition of TMDs to the minimal *2TM* structure, such as *6TM*. The pore-forming region (*P*) of GAAP is indicated in purple. Ion channel examples for the different types of structural motifs are given. The dotted lines link the most closely related structures, thereby identifying the proposed structural precursors. IP_3_R, inositol 1,4,5-trisphosphate receptor.

Structurally, the simplest of all known prokaryotic and eukaryotic channels consists of two transmembrane segments (2TM) (figure 3, green), separated by a selectivity filter and pore-forming loop known as the P-region. This basic motif, which is adopted for instance by the eukaryotic K^+^ inward rectifier (Kir) and the prokaryotic K^+^ channel (KcsA), is thought to form the basic building block from which the diverse other ion channel types have evolved [74,80,81]. This structure has been expanded with the addition of four transmembrane (TM) segments (figure 3, blue) to form the 6TM arrangement (named S1–S6) of most eukaryotic channels, such as voltage-gated K^+^ channels (K_v_), and forms the structural basis for Na^+^ and Ca^2+^ voltage-gated channels (Na_v_ and Ca_v_, respectively) [80–82]. Typically, S4 contains the voltage sensor, while S5–S6 contain the selectivity filter. Duplication and fusion events using a combination of the 2TM precursor unit and the 6TM structure are thought to have therefore given rise to the larger channel structures shown in figure 3.

Although the evolution of K^+^ channels has been traced back to the prokaryotic world as a 2TM structure, phylogenetic studies have usually excluded ion channels of viral origin based on the assumption that viruses may have acquired host genes by horizontal gene transfer [74,80]. However, phylogenomic evidence that some viral genes, including viral K^+^ channel-encoding genes, did not originate from their hosts [83,84] has highlighted the importance of including viral ion channels in these evolutionary analyses. This is particularly relevant to large algae-infecting viruses such as chloroviruses, which lack aa similarity between viral and host proteins or of close orthologues in databases, thus raising the question of the true donor organism(s) of these genes [83,84]. With the subsequent inclusion of viral K^+^ channels in these evolutionary studies, viral ion channels with the 2TM configuration, such as the chlorella virus Kcv channel, have been added to the list of progenitor-like channels (figure 3) [85]. These chloroviruses-encoded K^+^ channels have even been hypothesized by phylogenetic analysis as the closest channels to the evolutionary ancestor of all K^+^ channel proteins [85], consistent with evidence that some large DNA viruses may have predated or coexisted with the last universal common ancestor of bacteria, archaea and eukarya [86–88].

**Figure 4.**
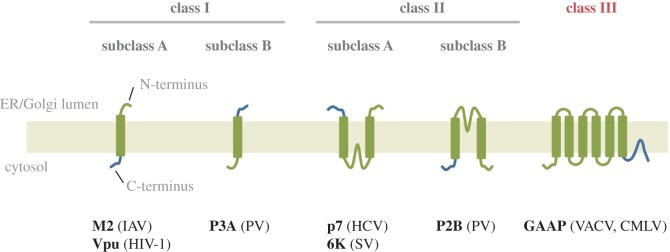
Topology-based classification of viroporins/viral ion channels. Class I viroporins contain proteins with a single transmembrane domain that are inserted into the membrane with either a lumenal N terminus and cytosolic C terminus (class IA) or the reverse orientation (class IB). Class II viral pores contain two transmembrane domains. Members of subclass A have lumenal N and C termini, whereas members of subclass B have cytosolic N and C termini. Examples of known viroporins of each subclass are shown. An additional class (III) is proposed for viral pores containing 6–7 transmembrane domains, which do not fit within the conventional classes I or II. HCV, hepatitis C virus; IAV, influenza A virus; HIV-1, human immunodeficiency virus 1; PV, poliovirus; SV, Sindbis virus; Vpu, viral protein U.

The first described viral ion channel is the M2 protein from influenza virus A [89]. Since then, other ion channels encoded by viruses have been discovered and are grouped within a family of viral ion channels known as viroporins. These are typically much shorter than cellular ion channels encompassing between 50 and 120 aa and contain no more than 1–3 TM regions that homo-oligomerize often into tetramers, thus constituting minimalistic versions of ion channels [90,91]. For instance, the 97 aa M2 protein contains a single TM [92] that assembles into a homo-tetrameric H^+^-permeable pore important for viral entry [93,94]. Once the virus is taken into the endosome, acidification of the virion interior mediated by M2 promotes virus uncoating and the release of viral RNA into the host cell [95]. Another example is the 94 aa viral K^+^ channel, Kcv, from chlorella virus 1 (*Paramecium bursaria* Chlorella virus 1, PBCV-1) that resembles the 2TM bacterial channels Kir and KcsA [96,97]. The two putative TM regions of Kcv are separated by a 44 aa pore region that contains the TXXTXGFG signature pore sequence of K^+^ channels [97]. PBCV-1 induces the rapid depolarization of the infected cells, and this is believed to be the result of Kcv channel incorporation into the host membrane and mediating K^+^ efflux from the cell [98]. Other examples of viral ion channels include Vpu from human immunodeficiency virus-1, p7 from hepatitis C virus, VP4 from poliovirus and 3a from severe acute respiratory syndrome-associated coronavirus (SARS-CoV) (figure 4).

**Figure 5.**
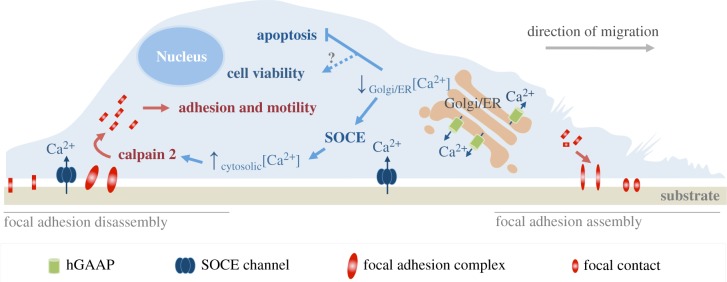
Summary model of the functional effects of GAAP in regulating intracellular ion flux, apoptosis, cell adhesion and migration. Modified from Saraiva *et al*. [7].

In contrast, the topology of GAAP is unlike any other viral ion channel described hitherto and contains some key differences with known eukaryotic or prokaryotic ion channels outside of the TMBIM/LFG family. GAAPs have short inter-membrane loops ranging from 3 to 11 aa, with the largest membrane-free region being a short cytosolic tail of approximately 35 aa at the N terminus [3,4]. Topology mapping, by the insertion of peptide epitopes into different inter-TM loops or at the N or C terminus of GAAP, indicated that GAAPs have both the N and C termini in the cytosol, 6 TMDs and an additional C-terminal hydrophobic region or loop (figures 1, 4 and 5) [3]. On the other hand, the high-resolution structure of YetJ from *Bacillus subtilis* (BsYetJ), a bacterial orthologue of hBI-1 and hGAAP (with about 20% aa identity) interpreted to form a H^+^-regulated Ca^2+^ channel, revealed a seventh TMD, located at the core of the structure [52]. The origin of the difference in the apparent organization of this hydrophobic region 7 between GAAPs and BsYetJ is not clear. It is possible that attaching a tag such as the hemagglutinin (HA) epitope, despite being short (9 aa), at the C terminus of GAAPs induced an aberrant topology, although GAAP tagged in this way retained its function as a regulator of apoptosis, adhesion, migration and Ca^2+^ homeostasis [3,4,7,8]. It is also possible that the C terminus of GAAP is in equilibrium between two states, one of which is favoured by the addition of a C-terminal tag and the other by the crystallization conditions used to solve the structure of BsYetJ, a related highly hydrophobic protein. Alternatively, there may be genuine differences in the membrane topologies of GAAPs and the distantly related hBI-1 bacterial protein.

The identification of vGAAP residues important for the pore (E^207^ and D^219^), their conservation among distantly related proteins and topological data suggest that the C-terminal hydrophobic loop probably lines the channel pore (figure 2) [4]. Therefore, GAAPs differ from the 6TM consensus of K^+^ channels, in which the pore region is located between TM5 and 6, in that the pore region of GAAPs is shifted further towards the C terminus (figure 3). Likewise, the crystal structure of BsYetJ is structurally different to any known ion channel [52]. Even in terms of size, 237 aa and 6–7 TM regions, GAAP is approximately threefold larger than that of any other viroporin (figure 4).

Considering that GAAP topology and the location of its pore region does not fit well within the current structural consensus of ion channel evolution, this suggests that GAAP may have evolved from a modified branch of the 2TM Kir-like precursor unit or from a different precursor (figure 3). GAAPs present a novel type or sub-type of ion channel structure, unlike any known viroporin in size, structural complexity and function (figures 3 and 4). Viral GAAP therefore represents a novel class (type III) of viral channels (figure 4).

The majority of viroporins have been associated with viral entry, assembly or release [99] and serve as ideal anti-viral drug targets. The same is true for many mammalian and prokaryotic channels implicated in disease pathology. However, issues relating to target specificity can often give rise to important side-effects and acquired drug resistance, as seen, for instance, with the M2-targeting compound amantadine [100,101]. Consequently, more detailed electrophysiological, structural and functional properties of channels are of particular value for improved targeted drug development.

## 3. Why GAAPs confer such broad-ranging protection against apoptotic stimuli

Considering that apoptotic regulators are mostly localized in the cytosol, the ER or the mitochondria, the Golgi represents an unconventional location within the cell from which to regulate apoptosis. In an attempt to address whether Golgi localization is required for its anti-apoptotic functions, a range of mutations (single aa substitution, short sequence changes, truncations and chimaeras) were introduced to alter GAAP localization. However, this has only yielded inconclusive results (unpublished data), probably reflecting the tight interplay between protein structure and function.

When compared with other TMBIM members, GAAP is the broadest anti-apoptotic inhibitor (table 1). Cells overexpressing vGAAP or hGAAP and challenged with a variety of intrinsic and extrinsic pro-apoptotic stimuli showed an increased resistance to programmed cell death [2]. However, the mechanism behind such a broad range of protection is unclear. Perhaps, given the central role of Ca^2+^ in apoptosis, it is likely that modulation of Ca^2+^ by GAAP plays a role in this process [4,6,7]. The fact that GAAP overexpression reduces the Ca^2+^ filling state of the ER and Golgi suggests that apoptosis protection may derive from reduced release of Ca^2+^ from intracellular stores upon pro-apoptotic stimuli, leading to a reduced entry of Ca^2+^ in the mitochondria and thus delaying and hampering apoptosis [6] (figure 5). The mechanism by which GAAP and other TMBIM family members control apoptosis requires further analysis and it is possible that the anti-apoptotic activity of these proteins constitutes a secondary effect of their regulation of Ca^2+^.

## 4. GAAP-mediated regulation of cell motility and adhesion

Several cellular processes are affected when TMBIM protein expression is manipulated. In addition to protecting cells from apoptotic stimuli and modulating the content of intracellular Ca^2+^ stores, overexpression of vGAAP and hGAAP increases cell migration, adhesion and spreading [4,7]. Store-operated calcium entry (SOCE) is enhanced by hGAAP overexpression and leads to greater activation of calpain 2 near the plasma membrane (PM), probably by binding free Ca^2+^ entering the cell from the extracellular space [7] (figure 5). Active calpain 2 accelerates the turnover of focal adhesions thereby contributing to the observed increased cell migration, adhesion and spreading phenotypes [7]. Conversely, the opposite phenotype is observed upon hGAAP knock down [7].

The effects of GAAP on cell motility are consistent with the described role of GAAP as a cation-selective channel [4]. When a residue that affects the ion channel conductance (E^207^) of vGAAP was mutated, the impact of protein overexpression on cell migration was lost, demonstrating the importance of the ion channel activity for GAAP-induced cell migration [4].

The detailed mechanism by which GAAP stimulates SOCE remains unclear. Considering the localization of GAAP within the Golgi and its ability to modulate the Ca^2+^ content of both the Golgi and the ER, several mechanisms are possible: (i) the depletion of luminal Golgi Ca^2+^ may affect the Ca^2+^ content of the ER and activate the typical SOCE pathway involving Orai1 and stromal interaction molecule 1 (STIM1); (ii) GAAP induces an alteration in Orai1 and/or STIM1 proteins; and (iii) GAAP within the Golgi contributes directly to SOCE activation via an unknown Golgi SOCE sensor(s). Positioning of the Golgi to the rear of the nucleus has been proposed to be important in regulating polarization and directed cell migration (reviewed in [102]). Whether or not hGAAP participates in Golgi positioning and polarization during cell migration by affecting the activation and/or localization of calpain 2, SOCE-related proteins or by any other mechanism is unknown.

Two other members of the TMBIM family (TMBIM6/BI-1 and TMBIM2/LFG) can also affect cellular mechanisms involved in cell migration and adhesion. Interestingly, increased expression of both BI-1 and LFG correlated with increased metastasis [36,63]. BI-1 overexpression induces cell migration by directly interacting with actin and by promoting actin polymerization [25]. Like GAAP, BI-1 induces SOCE, and this is dependent on the C-terminal lysine residues involved in actin binding [25], suggesting a possible link with cytoskeletal remodelling.

In a neuroblastoma (NBL) cell model, LFG repression resulted in reduced cell adhesion, increased sphere growth and enhanced migration [36]. Similarly, LFG knockdown increased the *in vivo* metastatic potential of SH-SY5Y and altered the expression profiles of several genes involved in cell adhesion and migration [36]. This supports a role for TMBIM members as cell motility regulators and possibly as players in tumour progression and metastasis.

## 5. Future perspectives

Despite much progress, it remains unclear whether the multiple functions of GAAP are linked or separate. The specific inhibition of ion channel activity by pharmacological inhibition or by mutagenesis could provide an interesting tool to dissect the mechanisms involved in each of GAAP's described functions. Data to date suggest that all GAAP-dependent functions rely on its ion channel activity, but contributions from the regulation of other Golgi ion channels or Golgi resident proteins remain possible. Generation of a GAAP knockout mouse (if viable) would shed some light on the important *in vivo* functions of GAAP as well as possible interactions with other TMBIM family members. All published TMBIM gene knockout mice are viable [41,47,103]. However, the importance of GAAP for cell viability increases the likelihood of lethality *in vivo*. Double TMBIM knockout mice lethality has also been suggested due to possible complementary functions of these proteins. Therefore, generation of conditional knockout mice in future will help to dissect the role of GAAP *in vivo* in homeostasis and disease models.

Although much is known about the roles of GAAP in the cell, the exact role for GAAP during poxvirus infection remains unclear. vGAAP expression by VACV strain Evans did not affect virus replication or spread in cell culture but reduced the virulence of this VACV strain following infection in mice [2]. The infection with a virus lacking vGAAP was characterized by enhanced infiltration of leucocytes into the infected tissue, showing that vGAAP is immunomodulatory. A comparison of four different anti-apoptotic proteins encoded by VACV showed that, in comparison, vGAAP is not a potent anti-apoptotic protein when expressed during viral infection, making it unlikely that its anti-apoptotic activity explains fully its effect on virulence [104].

TMBIM1, 2, 3 and 4 can all be found in Golgi membranes, but most of their role(s) in this organelle remain elusive. Shedding light on the processes in which these proteins are involved could help to dissect and understand Golgi functions. One of the central questions about the Golgi revolves around the spatial organization of the signals arriving at and originating from this organelle. How GAAP that is localized in the Golgi can activate Ca^2+^ entry from the extracellular space that occurs at the PM remains unclear. Golgi-originated stress signals and sensors involved in organelle-initiation of apoptosis have been proposed to mediate this, but no specific protein has been identified in this context thus far [105–107].

Given the fact that hGAAP can protect cells from apoptosis, promote cell viability, and upregulate cell adhesion and migration, it is possible that hGAAP has a role in tumour progression. Cell hyper-proliferation within confined, nutrient-poor environments triggers a variety of apoptotic stresses, thus anti-apoptotic genes are important contributors of cancer progression. In addition, activation of SOCE, calpain 2 activity, and alterations in the migration and attachment capabilities of cancer cells to other cells or the extracellular matrix are typical hallmarks of carcinoma progression to higher-grade malignancies [108–110].

**Figure 6.**
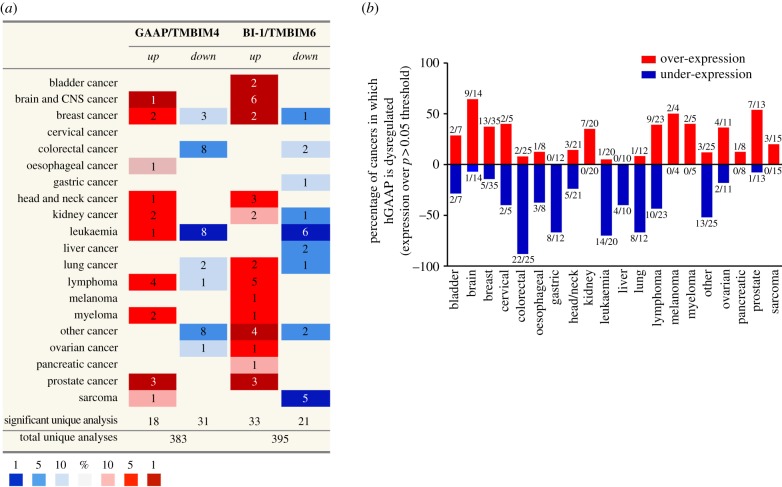
Global dysregulation of hGAAP mRNA in cancers. The Oncomine cancer microarray database (http://www.oncomine.com) was used to assess the dysregulation of hGAAP expression in several cancer tissues relative to healthy subjects. (*a*) Comparison of expression pattern between hGAAP and hBI-1. The table shows the number of studies reporting mRNA fold change ≥1.5 relative to normal tissue, using a threshold *p*-value of 0.05. The colour code reflects the best gene percentile (i.e. the top 1, 5 or 10% group of most altered genes). (*b*) Comparison of the frequency of hGAAP dysregulation between different cancer tissues. Data show the percentage of cancers in which hGAAP mRNA is over-expressed (red) or under-expressed (blue) compared with normal tissue, using a dysregulation threshold of *p* > 0.05. Numbers indicate the ratio between studies in which hGAAP mRNA was dysregulated, and the total number of studies investigating GAAP transcripts levels (e.g. out of the 13 studies that measured GAAP expression in prostate cancers, 7 significantly over-express hGAAP).

Significant upregulation of hGAAP mRNA has been detected in brain and lung tumours. In glioblastoma multiforme, high hGAAP mRNA levels are associated with poor outcome [43], and the dysregulation of hGAAP in non-small cell lung carcinoma samples led to hGAAP being proposed as a novel candidate prognostic marker for this disease in non-smoking patients [44]. An analysis of currently available microarray studies via the Oncomine platform indicates that hGAAP is over-expressed (figure 6*a*), with greatest frequency (figure 6*b*) in cancers of the brain and prostate, and under-expressed with unusual frequency in colorectal cancers (figure 6*b*). Unlike a previous report [18], this analysis showed that the pattern of hGAAP dysregulation among cancer tissues aligns with that of hBI-1, and this may become clearer as more studies include both BI-1 and hGAAP probes. BI-1 is also upregulated in some glioma [60] and lung cancers [59,61], as well as prostate [58,62] and breast cancer [56,57]. FAIM2/LFG/TMBIM2 overexpression also correlates with high primary breast tumours grades [38], and low LFG levels correlate with poor overall survival of NBL patients [36].

As GAAP confers resistance to a wide range of apoptotic stresses *in vitro*, including the anti-cancer drugs cisplatin and doxorubicin [2], it would be important to determine whether hGAAP correlates with resistance to chemotherapy and/or with poor prognosis, and thus represents an important indicator and therapeutic target.
